# General and Target-Specific DExD/H RNA Helicases in Eukaryotic Translation Initiation

**DOI:** 10.3390/ijms21124402

**Published:** 2020-06-20

**Authors:** Leo Shen, Jerry Pelletier

**Affiliations:** 1 Department of Biochemistry, McGill University, Montreal, QC H3G 1Y6, Canada; leo.shen@mail.mcgill.ca; 2 Department of Oncology, McGill University, Montreal, QC H4A 3T2, Canada; 3 Rosalind and Morris Goodman Cancer Research Center, McGill University, Montreal, QC H3A 1A3, Canada

**Keywords:** helicase, translation, initiation, DDX, DHX, eIF4A1, DDX3X, DHX9, DHX29, DHX36

## Abstract

DExD (DDX)- and DExH (DHX)-box RNA helicases, named after their Asp-Glu-x-Asp/His motifs, are integral to almost all RNA metabolic processes in eukaryotic cells. They play myriad roles in processes ranging from transcription and mRNA-protein complex remodeling, to RNA decay and translation. This last facet, translation, is an intricate process that involves DDX/DHX helicases and presents a regulatory node that is highly targetable. Studies aimed at better understanding this family of conserved proteins have revealed insights into their structures, catalytic mechanisms, and biological roles. They have also led to the development of chemical modulators that seek to exploit their essential roles in diseases. Herein, we review the most recent insights on several general and target-specific DDX/DHX helicases in eukaryotic translation initiation.

## 1. Introduction

RNA helicases play a role in all facets of RNA metabolism, ranging from transcription and translation, to processing and decay. These highly conserved enzymes use ATP to bind, unwind, and disrupt RNA structures and RNA-protein complexes [1]. Found in all domains of life, helicases are classified into six superfamilies (SFs), which are further sub-divided into families [1]. All eukaryotic RNA helicases belong to SFs 1 and 2, which comprise non-ring-forming structures [2]. Within SF2, the two largest families are the DExD-box (DDX) and DExH-box (DHX) helicases, named after their Asp-Glu-x-Asp/His signatures, which share evolutionarily conserved motifs within their core. 

Eukaryotic translation initiation is an intensively studied process and is subject to much regulation—either through the modulation of Met-tRNA_i_^Met^•GTP•eukaryotic initiation factor (eIF) 2 ternary complex availability or the flow of ribosome recruitment to mRNA templates by eIF4F [3]. This latter avenue requires the translation apparatus to successfully negotiate intricate mRNA secondary structures and stably bound proteins in order to establish a bona fide 80S complex at the initiation codon that is competent for protein synthesis. It is here that RNA helicases play a central role in the regulation of gene expression. Dubbed the “godfather” of DEAD-box helicases, eIF4A is a minimal DDX protein that consists of only the conserved ATP-binding and RNA-binding sites and short N- and C- terminal extensions (~50 and ~30 aa, respectively) [4]. It is the best-characterized RNA helicase in translation initiation and represents a prototypical DDX protein. Studies surrounding eIF4A have illuminated much of our understanding of the roles that DDX family members play, and many multi-faceted RNA helicases have since been explored.

In this review, we seek to outline the different RNA helicases that have been implicated in eukaryotic translation initiation (Table 1). We refer the reader to a previous excellent review on this topic [5], which this current piece seeks to update. First, we introduce the DDX helicase family and then provide an up-to-date summary on the biophysical and biochemical information surrounding its archetypal member, eIF4A. Then, we review recent information on other helicases that have been implicated in translation, some of which have been attributed a general role, and others a more mRNA- or structure-specific function. Due to space limitations, we restrict, for the most part, our discussions to the mammalian setting and to those helicases with a putatively direct role in translation initiation.

### 1.1. Translation Initiation and the Role of RNA Helicases

**Figure 1:**
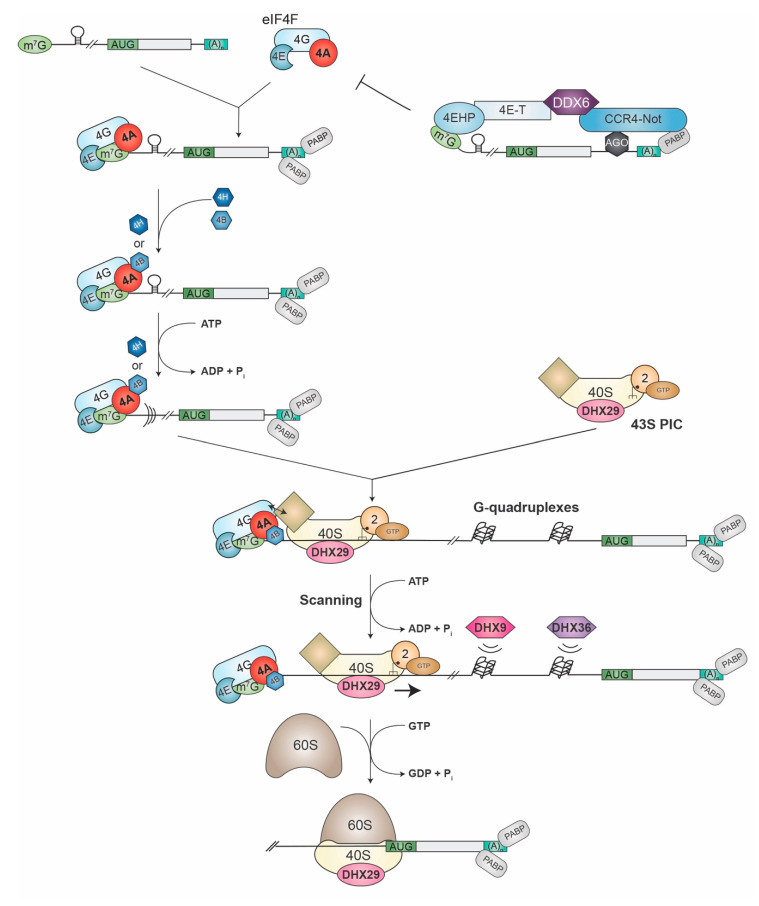
Schematic diagram of mammalian cap-dependent translation initiation pathway. See text for details. Please note for ease of viewing, the PABP•eIF4G bridging interactions are not shown. The diamond shape on the 40S ribosomes symbolizes eIF3, eIF1, eIF1A, and eIF5.

The recruitment of ribosomes to cytoplasmic mRNAs occurs by one of two mechanisms—cap-dependent and cap-independent processes. Cap-dependent translation is facilitated by the 5′-terminal m^7^GpppN (where N is any nucleotide) cap structure and is mediated by the eIF4F complex, which consists of eIF4E (the cap-binding subunit), eIF4G (a large scaffolding protein), and eIF4A (the ATP-dependent RNA helicase) (Figure 1). The RNA chaperones, eIF4B and eIF4H, interact with eIF4A, stimulate its activity (see below), and facilitate ribosome recruitment. An array of factors (the multi-subunit eIF3, eIF1, eIF1A, eIF2•GTP•Met-tRNA_i_^Met^, eIF5) bind to the 40S ribosomal subunit and convert it into the 43S pre-initiation complex (PIC). Subsequent interactions between eIF4G and eIF3 are critical for recruitment of the small ribosomal subunit to mRNAs. Once bound, the 43S PIC is thought to scan the 5′ leader region until an appropriate initiation codon is encountered, at which point initiation factors are evicted from the 48S complex, an additional molecule of GTP is hydrolyzed by eIF5B, and a 60S subunit is joined to the 40S to form an 80S complex now poised for elongation. This process is stimulated by the circularization of the mRNA, mediated by interactions between eIF4G and the 3′-end-bound poly (A) binding protein (PABP) [6]. Although the binding of eIF4E to the cap does not require ATP hydrolysis, the subsequent binding of eIF4A and eIF4B to the mRNA [7], as well as ribosome scanning [8], are ATP-dependent—a reliance that is thought to reflect the involvement of helicase activity in cap-dependent initiation. 

Cap-independent translation, mediated by internal ribosome entry sites (IRESes), on the other hand, have been stratified into four classes (types), with Types 1 and 2 requiring eIF4G and eIF4A, and Types 3 and 4 capable of recruiting ribosomes without the aid of any eIF4F subunits [3,9]. Moreover, initiation on some IRESes bypasses the requirement for scanning by directly situating the ribosomes at the initiation codon [3]. Helicase requirements for initiation are thus not equivalent among all IRES-containing mRNAs. 

A determinant of eIF4F activity is mRNA secondary structure. The secondary structure within 5′ leader regions has long been known to inhibit translation and, when located near the cap, to negatively impact the interaction of eIF4F and eIF4B with the mRNA [10,11,12,13,14]. Similarly, stable cap-proximal mRNA-protein complexes are also effective at suppressing translation [15]. The degree of secondary structure within an mRNA 5′ leader region links initiation of that mRNA to eIF4A activity. The greater the amount of structure, the more dependent that mRNA appears to be on eIF4A (and hence eIF4F) activity for initiation [14]. Unstructured synthetic mRNA templates are able to recruit ribosomes in the absence of ATP. However, this process is stimulated by eIF4A, and introducing even a weak stem-loop structure (ΔG = −6.7 kcal/mol) renders the reaction eIF4A- and ATP-dependent [16]. A class of cellular mRNAs containing very short 5′ leaders known as TISU (translation initiator of short 5′ UTR) elements are capable of initiating translation in an eIF4E-dependent, but eIF4A-independent manner [17,18]. TISU elements are common in mRNAs encoding mitochondrial proteins, which may have evolved to be less dependent on eIF4A [18]. Given the link between mitochondrial outer membrane integrity and apoptosis, this may be a way to buffer against large changes in eIF4A availability while remaining tuned to the eIF4E activity. Structure within the 5′ leader, therefore, is a critical determinant of translational efficiency—a notion that has been supported by large-scale ribosome footprinting studies, wherein eIF4A activity has been perturbed by small molecule inhibitors [19,20,21].

### 1.2. Structural Basis of Helicase Activity

**Figure 2:**
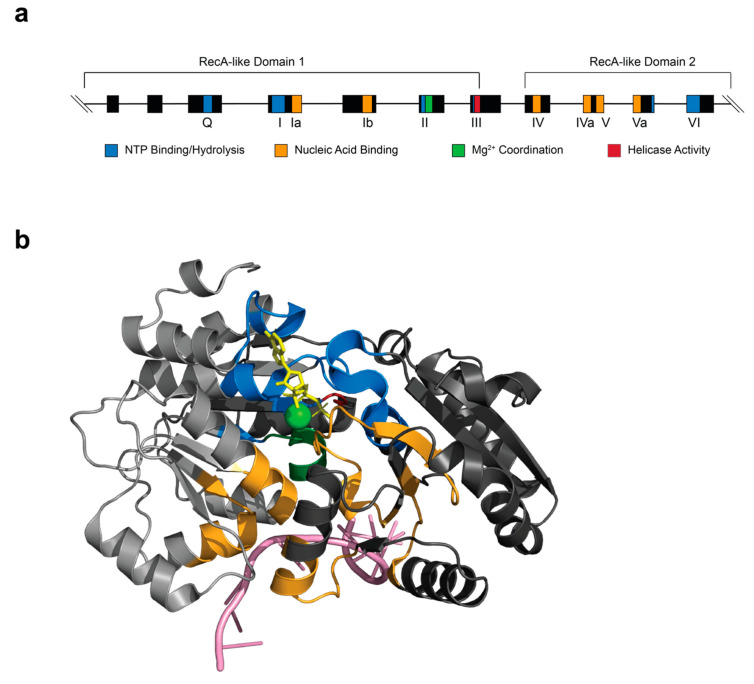
Structure of a conserved DDX/DHX helicase core and the archetypal eIF4A. (**a**) Exon-intron organization of the eIF4A1 gene with the location of the conserved domains highlighted. The functional role of each domain is denoted by a color code. (**b**) Three-dimensional structure of eIF4A complexed with AMP-PNP (yellow) and RNA (pink). The relative spatial location of the different functional domains is shown and color-coded as in (**a**). A magnesium ion is shown in green. The structure is from Protein Data Bank (PDB, 5ZC9).

DDX/DHX proteins harbor two recombinase A (RecA)-like domains tethered together by a short, flexible linker at their core (Figure 2) [2]. The helicase core has been further characterized to contain at least 12 conserved motifs, with motif II housing the DExD/H sequence. These motifs are evenly spaced and line the cleft of the “dumbbell”-shaped protein [22] (Figure 2). Between the two RecA-like domains lie the ATP- and RNA-binding sites whose functional significance has been extensively reviewed [22] (Figure 2a). Structural and biochemical studies have shown that the ATP-binding cleft in DDX proteins must be in a closed conformation to efficiently bind and hydrolyze ATP [23]. 

DDX helicases act as molecular switches that normally adopt an open conformation in the absence of RNA or ATP and close upon cooperative binding of the two molecules, which occurs via a two-step process [24,25]. This new, more rigid structure induces contacts between ATP and residues within motifs I, II, and VI (Figure 2b). Nucleotide specificity for adenine is conferred by the Q-motif through a conserved glutamine residue [26,27,28] (Figure 2). RNA-binding involves motifs Ia, Ib, IV, IVa, V, and Va (Figure 2). Communication between the RNA- and ATP-binding sites is thought to occur through motifs III, IV, and IVa [29] (Figure 2). In the closed conformation, a single-stranded (ss) RNA is bent such that duplex formation is unfavorable [25,30]. The sugar phosphate backbone of the RNA makes contact with residues of the helicase core, resulting in the kinking of the molecule [26]. The unwound RNA is then released, an event which may be coupled to ATP-binding in some cases and ATP hydrolysis in others [30,31]. In this manner, RNA and ATP stabilize the helicase core and, in the case of eIF4A, ATP stimulates RNA-binding and unwinding, and RNA stimulates ATP-binding and hydrolysis [24,26,27,32]. The unwinding mechanism of DDX helicases, however, remains distinct from those of other families. Rather than operating by a translocation or “threading” mechanism, DDX proteins facilitate local duplex unwinding by clamping onto the duplex region and pulling the strands apart [33,34]. Therefore, this unusual mechanism limits unwinding to only short RNA duplexes, 10–12 nucleotides in length (for eIF4A), at any given time [35].

Beyond the helicase core, most DDX/DHX helicases also contain N- and C-terminal extensions. EIF4A is an exception in that it has very short extensions. For the other DDX/DHX helicases, however, these extensions impart functional diversity to the family. Among some of the activities gained are modified RNA/DNA/protein-binding, oligomerization, and nuclease capabilities [1]. 

It is important to note that DHX proteins, despite being overall quite similar to DDX family members, exhibit significant differences in key residues within their conserved motifs [36]. DDX family members are generally ATP-specific, whereas DHX members can hydrolyze different NTPs—a feature attributed to the presence of the Q-motif in DDX family members, and its absence in their DHX counterparts.

## 2. General DExD/H-Box Helicases in Translation

### 2.1. eIF4A: A Minimal DDX RNA Helicase

EIF4A was one of the first DDX proteins discovered and represents the quintessential DEAD-box helicase due to its minimal structure that contains all the activities characteristic of the family but not much more (Figure 2) [4]. EIF4A is present in mammals as three paralogs: Human eIF4A1 (DDX2A) and eIF4A2 (DDX2B), which share 90% identity at the amino acid level, and the distant cousin, eIF4A3 (DDX48), which shares 66% identity to eIF4A1 [3]. While eIF4A1 is essential and plays an active role within translation initiation, eIF4A2 is generally less abundant, non-essential, and less well-characterized in its role [3]. In fact, suppression of eIF4A1 leads to an increase in eIF4A2 that, on a molar basis, should rescue the eIF4A1-deficient phenotype but does not [37]. Furthermore, the two proteins are differentially expressed, with eIF4A1 levels being higher in proliferating cells, and eIF4A2 expression being elevated in quiescent cells [38,39]. The third paralog, eIF4A3, is largely a misnomer, as it does not participate in translation initiation but rather in exon-junction complexes (EJCs) [40]. 

Similar to all other DDX helicases, eIF4A does not exhibit directional or processive helicase activity alone [41]. It unwinds ~10–12 base pairs/hydrolyzed ATP, with a turnover rate (k_cat_) of 0.01–0.05 per second [42]. The RNA-binding site has further been mapped to span 10–15 nts in length [43], and although RNA binding to eIF4A1 occurs with low affinity and generally independently of sequence, ssRNA-binding substantially stimulates ATPase activity in vitro [44,45]. In contrast, dsRNA binds weakly to eIF4A and does not significantly stimulate the ATPase activity [32,44]. In terms of substrates, eIF4A alone has been shown to unwind RNA/RNA, RNA/DNA, and DNA/RNA, but not DNA/DNA duplexes in vitro [46]. While the unwinding rate depends on the thermodynamic stability of the duplex substrate and not its length nor sequence, ssRNA tails on either end increase the rate by ~30% [46]. Furthermore, eIF4A1 and eIF4A2 have slight, inherent biases for binding polypurine-rich RNA sequences [47].

While it has been demonstrated that dependence on the eIF4A unwinding activity is directly proportional to the amount of secondary structure within the 5′ leader, whether all structural barriers play equivalent roles in determining eIF4A-dependency is not clear. Certain studies, such as that of Wolfe et al. [19] suggest G-quadruplexes (G4) to be key determinants of eIF4A-mediated translation. However, the very existence of these G4 structures is debated, as they have proven elusive in more recent studies [48,49]. Both Guo et al. [48] and Waldron et al. [49] used a combination of transcription stalling assays, dimethyl sulphate treatment, SHAPE analysis, and 7-deazaguanine incorporation to show that G4s appear to be largely unfolded in eukaryotic cells and dictate eIF4A-dependence to a lesser extent than classical stable hairpins. A subsequent study by Waldron et al. [21], using hippuristanol (an inhibitor of eIF4A that will be discussed further below), suggests that the secondary structure closest to the AUG start codon is most important in dictating eIF4A dependencies, but this result awaits biochemical validation. RNA pseudoknots are another example of complex intramolecular RNA structures and consist of two stem-loops in which half of one stem is intercalated between the half of the other. A pseudoknot structure is present in the 5’ leader region of human interferon gamma (IFNG) mRNA for example and thought to be a component of its regulation by the interferon-inducible protein kinase, PKR [50]. Whether this structure can be resolved by eIF4A, implicating eIF4A in a PKR regulatory circuitry, has yet to be determined and could present a novel regulatory loop. 

EIF4A2 has been implicated in translational repression and microRNA (miRNA) regulation through its interactions with the Ccr4-Not complex [51,52]. This complex is recruited to mRNAs via PABP, miRNAs, and RNA-binding proteins (RBPs), among others, when an mRNA is targeted for deadenylation and decay [53,54,55,56]. Meijer et al. [57] first demonstrated that eIF4A2 activity is required for miRNA-mediated mRNA destabilization. Then, the same lab reported that eIF4A2 may exert this function by becoming incorporated into the Ccr4-Not complex and inhibiting the CNOT7 deadenylation activity or repressing translation by binding to purine-rich sequences proximal to the AUG start codon [51,52]. These experiments implicating a repressive function for eIF4A2 are at odds with eIF4A2 being able to incorporate into the eIF4F complex, which plays a stimulatory role in translation [58]. Moreover, eIF4A2 is not essential for cell viability, and miRNA regulation is not impaired in its absence [59].

EIF4A does not function alone in translation initiation. As indicated above, eIF4B and eIF4H act as eIF4A cofactors to stimulate protein synthesis [60]. EIF4B stimulates the ATPase and RNA-unwinding activities of eIF4A [61], increases the coupling of ATP hydrolysis and helicase activity [62], confers directionality to eIF4A [63], helps recruit the 43S PIC to mRNA [64], and has even been reported to stimulate translation of mRNAs with complex 5′ leaders independently of eIF4A [65]. EIF4H and eIF4B share a common binding site on eIF4A, thus making the binding of these two cofactors mutually exclusive, but eIF4H exhibits many of the same effects as eIF4B when complexed with eIF4A [60].

Only ~5% of eIF4A is thought to reside within the eIF4F complex at one time, while the majority exists as the free form in cells [66]. However, eIF4F-bound eIF4A exhibits drastically altered biochemical properties. Upon recruitment of free eIF4A into eIF4F, eIF4A gains 5′→3′ directionality, for example [41]. Introduction of either human eIF4G_682-1105_ (middle domain of eIF4G sufficient to bind to eIF43, RNA, and eIF4A), eIF4H, or eIF4B alone enhance the ability of eIF4A to unwind by just under 2-fold [63]. However, addition of both eIF4G_682-1105_ and eIF4H, or eIF4G_682-1105_ and eIF4B, stimulated activity synergistically, with a greater increase being observed for the 4A/4G/4B combination (~100-fold) [63]. The resulting complex also became processive, with translocations occurring in discrete steps of 11 +/− 2 base pairs (roughly equal to one turn of an RNA double helix), as assessed by a single-molecule optical trapping assay [63]. Similar findings have been reported by others using gel-shift unwinding [60], real-time fluorescence [62], RNA pull-down [67], and single-molecule fluorescence resonance energy transfer (smFRET) assays [68]. 

The mechanism by which the ATP turnover rate increased was found to be through a joint stimulation of eIF4A by eIF4G and eIF4B of the open-closed conformational cycle of the helicase [68]. Both eIF4B and eIF4H increase the affinity of eIF4A or eIF4F for nucleotides and RNA, as well as the coupling of ATP hydrolysis with RNA unwinding [69]. Additionally, the presence of eIF4H or eIF4B conferred greater substrate specificity to eIF4F—namely, only substrates with at least a 20-nt ssRNA tail adjacent to the duplex and only RNA/RNA duplexes [70].

### 2.2. Regulation of eIF4A and Additional Roles

Beyond its interactions with other initiation factors, the eIF4A availability itself is dictated by factors such as the tumor suppressor programmed cell death 4 (PDCD4) protein. Structural studies have revealed that PDCD4 binds eIF4A and prevents the formation of the closed conformation, thus blocking RNA binding [71]. Under normal circumstances, the interaction between PDCD4 and eIF4A is subject to S6 kinase 1 (S6K1)- and S6K2-mediated phosphorylation, both of which are under control of the mammalian target of rapamycin complex 1 (mTORC1) [72,73]. PDCD4 phosphorylation leads to its rapid degradation, thus linking mTORC1 regulation to the eIF4A:PDCD4 interaction [72,73]. Additionally, mitogenic signals may be transduced via eIF4G (e.g., through the phosphorylation of its HEAT-1/2 interdomain linker) and eIF4E (e.g., via eIF4E-binding proteins [4E-BPs]), which are also under mTOR regulation) to modulate eIF4A helicase activity indirectly [3].

The eIF4A activity is also regulated by the long non-coding (lnc) RNA, human brain cytoplasmic RNA 1 (aka BC200), a 200-nt, predominantly cytoplasmic RNA linked to neurodegeneration and cancer initiation and progression [74]. BC200 is overexpressed in a number of cancer types yet is undetectable in corresponding normal tissues [74]. A number of initiation factors have been found to interact with BC200 and its mouse homolog BC1, including PABP, eIF4A, and eIF4B [74]. Binding of PABP and eIF4B to BC200/BC1 is thought to lead to suppression of translation by sequestering these factors, thereby preventing their participation in initiation [74]. Interaction of BC200 with eIF4A uncouples ATP hydrolysis from the helicase activity, leading to a reduction in cap-dependent translation [75,76]. BC1 also colocalizes with dendritic mRNAs and polysomes, raising a potential role in localized inhibition of translation [77]. 

With regards to modified or additional roles of eIF4A, a recent study by Sokabe and Fraser [78] suggests the possibility of eIF4A participating in mRNA recruitment to the ribosome in a helicase activity-independent manner. Through fluorescence-based anisotropy assays, they showed that eIF3j, which binds anti-cooperatively with RNA to the A-site of the 40S ribosomal subunit, can have its affinity reduced by the addition of eIF4A and ATP alone [78]. This reduction was achieved in a helicase-independent manner, and the authors showed that eIF4A can use its ATPase activity to control the conformation of the 43S PIC [78]. This study is consistent with a subsequent investigation by Yourik et al. [79] using yeast eIF4A, which showed mRNA recruitment to the ribosome to be independent of the 5′ leader structural complexity.

The functional diversity of eIF4A was recently expanded by Tauber et al. [80], who described the modulation of RNA condensation in stress granules by the DDX protein. Specifically, Tauber et al. showed that eIF4A limits stress granule formation by acting as an RNA chaperone to prevent aggregation [80]. DDX proteins have previously been described to localize to stress granules in mammals, yeast, and bacteria [81,82,83]. The authors showed that eIF4A prevents intermolecular RNA-RNA interactions and condensation within stress granules via its RNA-binding activity [80]. They also demonstrate that although the RNA-binding activity of the DDX protein alone is enough to decrease RNA condensation, its ATP hydrolysis activity increases the effect by allowing multiple cycles of RNA binding [80].

### 2.3. DDX3X

DDX3, or Ded1p in yeast, is ubiquitously expressed and is involved in a plethora of biological processes. DDX3 can be considered the next-most-investigated DDX family member, after eIF4A [84]. Ded1p is essential in yeast and shares a high degree of homology with human DDX3 [85]. There are two DDX3 subfamily members: DDX3X, which is X-linked (Xp11.3-11.23), and DDX3Y, which is found on the Y chromosome and expressed predominantly in the testes [86,87]. Both proteins have longer N-and C-terminal extensions than eIF4A [84]. DDX3 members maintain RNA-dependent ATPase activity and ATP-dependent RNA helicase activity, but the RecA-like domains 1 and 2 alone are insufficient for full ATPase activity, suggesting that the sequences flanking the helicase core contribute to this function [88,89]. A crystal structure of a DDX3X-AMP complex and subsequent functional characterization have revealed that DDX3 harbors a unique sequence (10 nts: Residues 250–259) between motifs I and Ia, which is involved in nucleic acid binding, hydrolysis, and unwinding [88,89]. In contrast to many other DDX family members, DDX3 has weak specificity for NTP and sugar binding, yet many of the conserved interactions, including the Q-motif with nucleotides, are maintained [90]. The roles of DDX3/Ded1p are myriad and have been comprehensively reviewed [84,85]. Beyond translation, DDX3/Ded1p appear to function in nucleocytoplasmic shuttling [91], as a host factor in viral infection [92], in chromosome segregation during mitosis [93], and even in EJC and spliceosome association [94]. DDX3/Ded1p subfamily members exhibit stunningly diverse functions.

In translation, conflicting reports have found that DDX3X in HeLa and Huh7 reporter cell assays stimulate cap-dependent translation [95,96] but can also inhibit translation upon ectopic expression in Huh7 cells [97]. This dual role as a stimulator and repressor of translation has been suggested by Hilliker et al. [98] to be due to Ded1p/DDX3’s ability to form a Ded1p-mRNA-eIF4F complex (via Ded1p•eIF4G1 interactions) that is sequestered to stress granules but is then released following ATP hydrolysis by Ded1p. Microscopy experiments showed the formation of a stalled mRNP that is resolved by Ded1p’s ATPase activity [98]. This was also supported by the finding that Ded1p overexpression leads to an increased processing body (P-body) formation, where Ded1p further accumulates [99]. Several groups have also reported that DDX3X promotes the translation of a subset of mRNAs, including cyclins and transcripts with longer, more structured 5′ leaders [100,101,102]. Ribosome footprinting experiments with temperature-sensitive mutants of Ded1p and eIF4A showed that Ded1p-dependent transcripts harbor more cap-distal stem loops in their 5′ leaders and greater overall structure than eIF4A-dependent mRNAs [102]. The emerging picture from yeast is that eIF4A is required for translation initiation on all mRNAs, whereas Ded1p is needed to resolve 5’ leader structural barriers [102]. Furthermore, Ded1p also plays a key role in near-cognate initiation codon recognition—when Ded1p activity is repressed or diminished, the scanning ribosome initiates at near-cognate initiation codons that are within the 5’ leader and immediately upstream of the secondary structure [103]. A recent report has documented a role for Ded1p as a stress sensor that responds to environmental changes [104]. At elevated temperatures (>39 °C), Ded1p condenses/aggregates, an event that is associated with a repression in translation of mRNAs encoding housekeeping functions [104]. A role for DDX3X and DDX3Y in mammalian translation is less clearly defined and necessitates further investigation.

### 2.4. DHX29

DHX29 binds to 40S ribosomes through its N-terminal domain [105]. Its NTPase activity is strongly stimulated by 40S ribosomes, is required for efficient 48S complex formation, and plays a role in promoting translation initiation on mRNAs with elevated 5′ leader secondary structure [106]. It is present in 48S complexes but not polysomes, indicating that it recycles during the initiation of translation [107]. CryoEM studies have placed DHX29 at the tip of helix H16 of the 18S ribosomal RNA, with its distal part extending along the mRNA entry channel [108]. DHX29 promotes unwinding of stable stems and ensures a linear nucleotide-by-nucleotide scanning during initiation; in its absence, stem-loops enter the mRNA-binding channel but are not threaded through the exit channel [109]. It has been suggested that DHX29 could be responsible for closing the mRNA entry channel latch following slotting of the mRNA into the mRNA-binding cleft. This would trap the mRNA inside the channel and contribute to an increased efficiency of 43S scanning [108]. On its own, DHX29 does not possess processive helicase activity [106]; rather, recent data have shown that it makes contact with 40S-bound eIF3 subunits, which may implicate it in the rearrangement of the ribosomal complex and higher processivity in mRNA unwinding during scanning [110,111]. A consequence of this remodeling may explain the ability of DHX29 to suppress leaky scanning through upstream AUG codons (but not through non-cognate CUG codons), where DHX29 would rearrange the positioning of eIF1A and eIF2α (indirectly), thereby impacting AUG recognition [112]. 

Depleting DHX29 by RNAi disrupts polysomes, suppresses translation, and inhibits cancer cell proliferation [107]. Whether DHX29 is a viable anti-cancer target whose inhibition could achieve a workable therapeutic index remains to be explored. However, CRISPR/Cas9-mediated knockout of DHX29 in human and mouse non-transformed, primary cells is also lethal, indicating that DHX29 is an essential gene [113]. A role for DHX29 beyond translation has recently been reported, where it was shown to recognize double-stranded RNA and specifically interact with MDA5 to enhance innate antiviral immunity [113]. 

## 3. Target- or Structure-Specific Helicases

A number of less well-studied DDX/DHX-box helicases have also been implicated in translation. Although the evidence for these playing a role in the initiation process is mounting, biochemical and mechanistic insights are required to consolidate their role in this process and to properly situate where they stand in the initiation pathway (Table 1). Here, we review the DDX/DHX helicases currently implicated in the translation of specific mRNA species.

### 3.1. DHX36

DHX36 (aka RHAU or G4R1) binds DNA and RNA G4 structures with a high affinity *in vitro* and *in vivo* and is capable of resolving such structures [114,115,116]. It has been implicated in transcriptional and post-transcriptional regulation of gene expression, with essential roles in heart development, hematopoiesis, and embryogenesis [117,118,119]. A role for DHX36 (RHAU and G4R1) in the selective translation of mRNAs encoding mixed lineage leukemia (MLL) 1 and MLL4—pivotal leukemogenic transcriptional regulators—has been documented, whereby the Aven RNA-binding protein recruits DHX36 to G4 sequences [120]. Depletion of DHX36 in HEK293 cells does not impair global protein synthesis, but does result in a shift of both MLL1 and MLL4 mRNAs into lighter polysomes [120]. A specialized role for DHX36 in regulating expression of G4-containing mRNAs was obtained from genome-wide studies undertaken in HeLa cells, which showed that: (i) mRNAs with G4 structures in their 5′ leader regions are inefficiently translated, (ii) stable G4s can promote the translation of upstream open reading frames (uORFs) when positioned downstream of the uORF initiation codon, (iii) DHX36 (and DHX9) are polysome-associated, and (iv) suppression of DHX36 and DHX9 results in an impairment of translation of mRNAs harboring G4s in their 5′ leader region. Overall structure within the 5′ leader region was not a determinant of DHX36-responsiveness, although length was [121]. The consequences of DHX36 knockdown in HEK293T cells were also analyzed at the proteomic level, where only 1.9% of the identified proteins (1837 sampled total) were downregulated by at least 70% [122]. Among the suppressed targets, 33% were encoded by mRNAs containing G4s in their 5′ leader regions [122]. 

Co-crystallization of DHX36 with a DNA G4 structure has revealed that DHX36 interacts with both the top face of the G4 structure and the adjacent single-stranded nucleic acid segment [123]. DHX36 does not discriminate between DNA and RNA substrates, as its contacts with the nucleic acid backbone are primarily with phosphates [123]. The drosophila DHX36 homolog has been shown to stabilize G4s in the absence of nucleotide or when bound to AMP-PNP or ADP, but resolves said structures in the presence of ATP [124]. The domains interacting with both the G4 structure and 3′ tail are necessary for this destabilization [124]. 

DHX36 can also associate with the BC200 lncRNA [125]. This interaction is not G4-mediated but rather occurs through a 3′-end, adenosine-rich region [125]. Given the interaction between eIF4A and BC200, it would be interesting to investigate whether BC200 co-regulates the activity of eIF4A and DHX36. Whether and how the activity of DHX36 is coupled to core translation initiation factors and if it can be regulated in an mRNA-specific manner remains open to questions. 

### 3.2. DHX9

DHX9 has been implicated in a number of cellular processes ranging from DNA replication, transcription, translation, RNA processing and transport, miRNA processing, and maintenance of genomic stability [126]. A role for DHX9 in mRNA-specific translation has been documented, with implicated roles in the expression of viral and JunD mRNAs harboring a 5′ post-transcription control element (PCE)—a complex structural feature consisting of two stem-loops [127]. The NTPase/helicase activity of DHX9 is required for this function [128], and association with PCE-containing mRNAs occurs in the nucleus and cytoplasm, which may reflect an early post-transcriptional mark that serves to ensure efficient routing of the tagged mRNA into polysomes [128,129]. JunD mRNA translation is cap-dependent [130], and the possible interplay between eIF4F and DHX9 would be intriguing to explore. Bioinformatics analysis has identified ~200 human genes containing PCEs [131]. 

DHX9 facilitates the translation of α1 and α2 type I collagen, but in this case, via a different mechanism. The 5′ leader regions of the α1(I) and α2(I) mRNAs both harbor a stem-loop to which the La ribonucleoprotein (LARP) 6 family member binds and recruits DHX9, stimulating ribosome loading and translation initiation [132]. Knockdown of DHX9 in human lung fibroblasts prevents collagen mRNAs from loading into polysomes without affecting mRNA levels [133]. Similar to PCE-containing mRNAs, the binding of transcripts by LARP6/DHX9 occurs in the nucleus, prior to the resulting stimulation in translation [133].

DHX9 has also been implicated in IRES-mediated translation—specifically, of an IRES in the 5′ leader region of the p53 mRNA that is responsive to DNA damage. DHX9 and translational control protein (TCP) 80 cooperatively bind to the p53 IRES in response to exposure of cells to DNA-damaging agents [134,135]. The presence of DHX9 is thought to stimulate an IRES-mediated initiation event, leading to an increased translation of the p53 mRNA. 

*In vitro*, DHX9 has also been reported to bind and resolve RNA G4 structures [136]. Similar to DHX36, DHX9 is also found to be polysome- and G4-associated, with the translation of mRNAs harboring G4s being most sensitive to DHX9 knockdown [121]. Depletion of DHX9 in HeLa cells does not lead to a global inhibition of translation, and the spectrum of mRNAs inhibited somewhat overlapped with those responsive to DHX36 suppression (Pearson correlation = 0.56) [121]. Individual-nucleotide resolution UV crosslinking and immunoprecipitation (iCLIP) experiments revealed G4 sequences to be enriched for, and located ~40 nts upstream of, the DHX9 peak centers, possibly reflecting the pre-resolution of the G4 sequence with the helicase loading downstream of the structure [121]. Taken together, it appears that G4s are key determinants of DHX9 (and DHX36)-responsiveness. 

### 3.3. DHX33

DHX33 is not well-studied and was initially implicated in rRNA biogenesis [137]. However, its suppression in several cell lines led to a reduction in polysomes and a concomitant increase in monosomes—not what would be expected for an inhibitor of rRNA synthesis, but rather more akin to what one would expect for a block in initiation or disruption of polysomes [138]. Consistent with this, DHX33 knockdown reduces translation as assessed in ^35^S-Met metabolic labelling and luciferase reporter assays [138]. Sub-cellular fractionation studies have shown that although the majority of DHX33 is nuclear, a significant proportion is cytoplasmic [137,139]. DHX33 co-sediments with monosomes, but not polysomes, in sucrose gradients and can be found in pull-down experiments to be associated with eIF3g and several ribosomal proteins (rpL27, rpL26, rpL7 (but not rpS2)) [137,139]. DHX33 helicase activity was not required for this association, nor were the associations RNA-mediated (since they were not affected by RNAse treatment) [139]. *In vitro*, DHX33 failed to stimulate translation of a firefly luciferase reporter mRNA, indicating it may not be limiting in reticulocyte lysates, or that its role in translation *in cellula* is indirect [139].

DHX33 is highly expressed in several cancer types, including glioblastoma [140]. Knockdown in the U118-MG glioblastoma line resulted in reduced cell proliferation, cell migration, and/or reduced anchorage-independent growth [140], a result that is interesting, since it appears that DHX33 is not essential in most cell lines (https://oncologynibr.shinyapps.io/drive/). Overexpression of DHX33 conferred resistance to the mTOR inhibitors, rapamycin, and Torin1 [140].

### 3.4. DDX1

DDX1 was found to be an insulin mRNA-binding protein in RNA pull-down assays [141]. Prolonged exposure to free fatty acids impairs insulin secretion from pancreatic cells, and DDX1 is thought to mediate the palmitate-induced inhibition of insulin mRNA translation [141]. Palmitate treatment of cells causes DDX1 to re-localize from the cytoplasm to the nucleus. Knockdown of DDX1 results in a significant reduction in proinsulin levels with no change in insulin mRNA levels, whereas ectopic overexpression of DDX1 increases proinsulin protein levels (with mRNA levels remaining unchanged) [141]. DDX1 suppression causes insulin mRNA to shift into lighter polysomes, while palmitate treatment of cells leads to phosphorylation of DDX1 at S295, a modification that affects its association with insulin mRNA and its ability to rescue shDDX1-mediated suppression [141]. Immunoprecipitation experiments to identify interacting partners indicated an association with the core translation factors, eIF4B, eIF3A, eIF3B, eIF3M, eIF4G1, and eIF4G2 [141]. DDX1 was also found in a complex with hCLE, HSPC117, and FAM98B, in which hCLE was reported to have cap-binding activity [142]. Suppression of hCLE reduced the incorporation of ^35^S-Met into cellular proteins without impacting global mRNA levels. Whether and how hCLE specifically and directly recognizes cap structures awaits structural resolution. In pull-down experiments, 144 mRNAs were found to interact with hCLE in HEK293 cells [142]. Whether DDX1 plays a role in selecting target mRNAs remains to be established. 

### 3.5. DDX41

DDX41 was postulated to have a role in translation following its identification in RNA pull-down assays using the *in vitro* synthesized 3′ leader region of p21 (cyclin-dependent kinase inhibitor) mRNA [143]. Suppression of DDX41 led to elevated p21 protein levels with no change in mRNA levels, indicating that DDX41 negatively regulates p21 expression [143]. The helicase activity of DDX41 is essential to its ability to suppress p21 expression [143]. What is now needed are experiments providing mechanistic insight into DDX41-mediated translational control. 

### 3.6. DDX6

DDX6 has been implicated in mRNA storage, decay, and the repression of translation [144]. It has been shown to contribute to the translational repression exerted by miRNAs. The silencing activity of miRNAs is mediated by the Ccr4-Not deadenylase complex, with DDX6 and 4EHP (aka eIF4E2) also present as components of the miRNA-induced silencing complex (miRISC) [145]. 4EHP is a cap-binding protein that unlike eIF4E, does not interact with eIF4G, and its binding to the cap has been associated with a repressive function [3]. The C-terminal RecA-like domain of DDX6 interacts with the CNOT1 MIF4G domain in a manner reminiscent of the eIF4G-eIF4A complex [146]. DDX6 tethers 4E-T (eIF4E transporter) [147], which in turn can interact with eIF4E or 4EHP [148,149]. When 4E-T is bound to eIF4E, this enhances the decay of mRNAs targeted by the Ccr4-Not complex, including miRNAs [149]. The binding of 4E-T to 4EHP increases the affinity of 4EHP for the cap and engenders a closed-loop complex with 4EHP bound to the cap and DDX6 bound to Ccr4-Not residing at a miRNA-binding site (Figure 1). Since 4EHP cannot interact with eIF4G, this complex cannot recruit ribosomes and thus prevents eIF4F from initiating on the cyclized mRNA template [148]. In this manner, DDX6 is able to coordinate the repression of specific mRNA templates.

DDX6 has also been implicated in the stimulation of translation of specific mRNAs. In this case, ectopic expression of DDX6 resulted in elevated levels of c-Myc protein in COS-7 and SW480 human colorectal cells [150]. Translation of the c-Myc mRNA is cap-dependent and eIF4E-responsive [151], but it may also harbor an IRES [152]. DDX6 is thought to be an IRES-trans-acting factor (ITAF), where its helicase activity might resolve structural elements within the c-Myc IRES—an event that would be required for initiation of c-Myc [153]. In contrast, DDX6 has also been implicated as a repressor of the vascular endothelial growth factor (VEGF) IRES [154]. The VEGF IRES confers sustained translation under conditions of hypoxia. Recombinant DDX6 inhibited translation of VEGF mRNA *in vitro*, DDX6 levels decline during hypoxia, and DDX6 depletion stimulated DDX6 expression under hypoxia [154]. In this scenario, DDX6 would rearrange the VEGF IRES and render it non-functional under normoxic conditions. These contrasting activities of DDX6 might depend on a select suite of binding partner(s) that intimately regulate DDX6 activity while imparting mRNA binding specificity.

## 4. Targeting DDX/DHX-Box Helicases in Translation Initiation

Protein synthesis is the most energetically expensive process in cells, and consequently, its regulation or dysregulation will have profound implications on cellular physiology. Ranging from cancer biology to viral replication, perturbation of translation is found in many diseases. In particular, cancer (aberrant proliferation, angiogenesis, changes in the immune system, etc.) has been tied to altered translation initiation, the rate-limiting step of the entire process (reviewed in [155,156]). EIF4E overexpression, for example, is sufficient for neoplastic transformation and tumorigenesis both *in vitro* and *in vivo* [157,158,159]. Additionally, viruses, a subject of topical interest, also rely on the translation machinery and DDX/DHX helicases for their replication [160,161,162]. Beyond perturbing the translation process, however, diseases such as cancer and viral infection often develop a dependence on altered mRNA translation, raising the possibility that by targeting protein synthesis, one could drug this vulnerability [155,156]. For example, many oncogenes harbor long, highly structured 5′ leaders that exhibit a greater dependence on eIF4A helicase activity [19]. It has also been demonstrated that the translation of these mRNAs with complex 5′ leaders is directly proportional to the amount of eIF4A activity [14]. By targeting this therapeutic window, wherein translation of highly structured mRNAs (many of which encode for oncogenic functions) will be disproportionately affected compared to normal, non-oncogenic transcripts, one might be able to treat the disease without harming the individual [155,156]. The same can be said of viral infections, where the viruses’ replication depends more on host translation than do most of our regular homeostatic mechanisms. 

To date, several small molecules have been identified that selectively target DDX/DHX-box helicase family members, and these have been extensively reviewed in [163] (summarized in Table 2). In brief, helicase inhibitors can be stratified into two groups: (i) interfacial inhibitors, in which the compounds interact with both the protein target and the RNA, and (ii) compounds that inhibit RNA binding. Rocaglates and Pateamine A (and analogs) are interfacial inhibitors that cause eIF4A to clamp onto RNA, significantly stabilizing the resulting complex [164,165]. This gain-of-function produces a number of effects on translation initiation that inhibits the pathway through several modes: (i) clamped eIF4A molecules on 5′ leader regions inhibit scanning, (ii) eIF4F becomes clamped at the cap resulting in a reduced 43S pre-initiation complex loading, and (iii) stabilization of eIF4F onto RNA diminishes the pool of eIF4F and exerts an effect *in trans* on initiation [166]. Furthermore, eIF4A appears to also become clamped to ribosomes, the functional consequence of which remains to be investigated [167]. Interfacial inhibitors represent a powerful approach through which to inhibit a biological process such as translation, since only a small fraction of eIF4A molecules (enough to impose a steric blockade) needs to be engaged by the small molecule. Specificity of binding, at least for rocaglates where this has been defined through structural studies [164], is conferred by interacting amino acids (F163 and Q195) only found at these locations in eIF4A [164]. 

On the other hand, hippuristanol and 1,4-diacylpiperazine derivatives, respectively, target eIF4A1/2 and eIF4A3 by binding to the CTD and inhibiting RNA binding [168,169]. It appears from NMR studies that both compounds share fairly conserved binding pockets. Variomics screens undertaken with both compounds have clearly indicated that these are not pan-helicase inhibitors and have linked their biological effects to target engagement [170] (J. Pelletier, unpublished data). 

Two inhibitors of DDX3X have recently been reported [171,172]. RK-33 is a ring-expanded nucleoside that directly interacts with DDX3X and inhibits its ATPase and helicase activity. Given that DDX3X has been implicated in the replication of a number of viruses, RK-33 also shows broad-spectrum anti-viral activity [67]. Takeda also recently identified a new inhibitor of DDX3X helicase activity, C1 [172]. We envision that if these compounds can be shown to be selective, they will be useful in better defining the role of DDX3X in translation and its other associated processes.

Twenty-six compounds emerged from a high-throughput screen undertaken by Takeda for DDX41 ATPase inhibitors [173]. Their counter-screens probed for activity against DDX48 and DnaK, and they ensured that primary hits did not have RNA intercalation activity (which could non-specifically inhibit helicase activity). Two compounds that emerged from this screen (Compounds #1 and 2) are thought to inhibit nucleic acid substrate binding and act as non-competitive inhibitors of ATP binding [173]. 

## 5. Summary and Outlook

There is emerging interest in the study of RNA helicases, given their fundamental role in a number of biological processes. Even with the best-studied DDX helicase, eIF4A, there is much that remains to be uncovered with respect to its role in translation alone. Why is only ~5% of eIF4A present in the eIF4F complex? What role, if any, does the remaining 95% free eIF4A play in or outside of translation? It is becoming evident that one key to understanding helicase specificity of action is defining their interactomes, as their associated factors often impact function and modulate activity in drastic manners. Helicases also exhibit diversity in activity—from resolving RNA secondary structure, to affecting the processivity of the scanning ribosome, to remodeling protein complexes. How these activities are regulated is another aspect that remains to be explored. 

Lastly, the characterization of small molecules that can selectively interfere with helicase activity has opened up the possibility of targeting these for drug development and provided a roadmap for effectively inhibiting downstream gene expression. Due to their central role in many disease processes, RNA helicases represent a biological target with significant, unexplored potential. Whether it be targeting host helicases in cancer or viral helicases in infection, small molecules and therapeutics can only be further developed if we continue to expand on our knowledge.

## Figures and Tables

**Table 1:** RNA Helicases Implicated in Translational Control.

Helicase	Family	Proposed Mechanism of Action	Comments
DDX2A/DDX2B (eIF4A1/eIF4A2)	DExD	Unwinds the 5′ leader structure as part of the eIF4F complex; reduces affinity of eIF3j for the ribosome to enable the mRNA to access the entry channel.	eIF4A1 is essential; eIF4A2 is non-essential.
DDX3X	DExD	Ded1p homolog in yeast resolves a wider range of (more complex) 5′ leader structures than eIF4A. May interact with eIF4F and mRNA to modulate participation in stress granules.	Ded1p is essential in yeast; has been implicated as a stimulator and repressor of translation in mammals.
DHX29	DExH	Rearrangement of 43S complexes leading to higher processivity in unwinding during scanning.	
DHX36	DExH	Resolution of 5′ leader G-quadruplexes.	
DHX9	DExH	Resolution of 5′ leader G-quadruplexes. Resolution of PCEs and 5′ leader secondary structure.	
DHX33	DExH	Interacts with eIF3g and several ribosomal proteins.	Modifier of response to mTOR inhibitors (rapamycin and Torin1).
DDX1	DExD	Implicated in translation of insulin mRNA.	
DDX41	DExD	Implicated in translation of p21 mRNA.	
DDX6	DExD	Part of the miRISC and inhibits translation through tethering of 4E-T and cap-bound 4EHP.	Generally, a repressor of translation.

**Table 2:** Current Selective Inhibitors of DDX/DHX Family Members Implicated in Translation.

Compound	Target	Proposed Mechanism of Action	Comments
Rocaglates	eIF4A1/2	Clamping of eIF4A via F163 and Q195 onto polypurine-rich sequences in the 5′ leader region.	One derivative, eFT226 (Zotatifin), is in Phase 1–2 clinical trials (https://clinicaltrials.gov/ct2/show/NCT04092673).
Pateamine A (and analogs)	eIF4A1/2	Clamping of eIF4A onto RNA.	
Hippuristanol	eIF4A1/2	Binds to the eIF4A CTD, blocks ATPase, helicase, and RNA-binding activity.	
1,4-diacylpiperazines (T-595, T-202)	eIF4A3	Binds to the eIF4A3 CTD, inhibits ATPase, helicase activity, EJC formation, and NMD.	
RK-33	DDX3X	Docks in the ATP-binding domain of DDX3X, thereby inhibiting helicase function.	
C1	DDX3X	Possible selective binding to the ATP-binding pocket of DDX3X; blocks helicase activity.	
Compounds 1 and 2	DDX41	Inhibits ATPase activity.	

## Abbreviations

4EHP	eIF4E Homology Protein
4E-T	eIF4E Transporter
CCR4-Not	Carbon Catabolite Repression—Negative On TATA-less
DDX	DExD-box
DHX	DExH-box
eIF	Eukaryotic Initiation Factor
IFNG	Human Interferon Gamma
IRES	Internal Ribosome Entry Site
G4	G-quadruplexes
HEAT	Huntington, Elongation Factor 3, PR65/A, TOR
iCLIP	Individual-nucleotide resolution UV crosslinking and immunoprecipitation
ITAF	IRES Trans-Acting Factor
LARP	La Ribonucleoprotein
Lnc	Long Non-coding
MIF4G	Middle domain of eIF4G
miRISC	miRNA-Induced Silencing Complex
mTORC1	Mammalian Target of Rapamycin Complex 1
MLL	Mixed Lineage Leukemia
PABP	Poly (A) Binding Protein
PCE	Post-transcription Control Element
PIC	Pre-initiation Complex
PDCD4	Programmed Cell Death 4
PKR	Protein Kinase R
RBP	RNA Binding Protein
RecA	Recombinase A
S6K	Ribosomal Protein S6 kinase
SF	Superfamily
smFRET	single-molecule Fluorescence Resonance Energy Transfer
TCP	Translational Control Protein
TISU	Translation Initiator of Short 5’ UTR
uORFs	Upstream Open Reading Frames
